# Falciform Ligament Lipoma: A Rare Cause of Chronic Epigastric Pain

**DOI:** 10.70352/scrj.cr.26-0241

**Published:** 2026-06-24

**Authors:** Takamune Yamaguchi, Takashi Kokudo, Henri Vuilleumier, Nermin Halkic

**Affiliations:** 1 Department of Surgery, Tokyo Metropolitan Bokutoh Hospital, Tokyo, Japan; 2 Department of Surgery, Hepato-Biliary Pancreatic Surgery Division, National Center for Global Health and Medicine, Japan Institute for Health Security, Tokyo, Japan; 3 Department of Surgery, Hirslanden Clinique Cecil, Lausanne, Switzerland

**Keywords:** falciform ligament, lipoma, laparoscopy, epigastric pain, abdominal neoplasms, perivascular epithelioid cell tumor, differential diagnosis

## Abstract

**INTRODUCTION:**

Lipomas of the falciform ligament are exceptionally rare benign tumors, with only 9 cases reported in the literature since 1983. These lesions can present with chronic abdominal pain and pose significant diagnostic challenges due to their rarity and overlap with other pathological entities.

**CASE PRESENTATION:**

We report a case of a 49-year-old female presenting with chronic epigastric pain radiating to the right hypochondrium. MRI revealed a large fatty mass measuring 9 × 2.7 × 8.5 cm in the falciform ligament region. The patient underwent successful laparoscopic excision with histopathological confirmation of a benign lipoma. Negative MDM2 immunostaining excluded liposarcoma, and negative HMB-45, smooth muscle actin, and melan-A immunostaining excluded perivascular epithelioid cell tumor (PEComa).

**CONCLUSIONS:**

Although exceptionally rare, falciform ligament lipoma should be considered in the differential diagnosis of chronic epigastric pain alongside other falciform ligament pathologies, including PEComas and liposarcomas. Complete laparoscopic excision represents the treatment of choice with excellent outcomes, although diagnostic laparoscopy may be required for anatomical localization.

## INTRODUCTION

The falciform ligament is a double fold of peritoneum that extends from the anterior abdominal wall to the liver, containing the ligamentum teres hepatis (round ligament) and variable amounts of fat tissue.^1)^ This anatomical structure marks the division between the left lateral and medial segments of the liver and represents an embryological remnant of the umbilical vein system.

Tumors arising from the falciform ligament are exceptionally rare, with lipomas being among the most uncommon pathologies affecting this structure.^1,2)^ According to the comprehensive literature review by Bangeas et al.,^3)^ only 7 cases of lipoma originating from the falciform ligament had been documented since 1983 at the time of their review, with their case representing the 8th reported case. Subsequently, Sadeghi et al.^4)^ reported the 9th case, making this condition one of the rarest entities in abdominal surgery.

The clinical presentation of falciform ligament lipoma is often nonspecific, typically manifesting as chronic abdominal pain that can mimic other intra-abdominal pathologies.^3,4)^ The extreme rarity of this condition often leads to delayed diagnosis and unnecessary investigations. Furthermore, the differential diagnosis has expanded significantly with the recognition of other rare falciform ligament tumors, particularly perivascular epithelioid cell tumors (PEComas), which have been increasingly reported in the recent literature.^5–7)^

The imaging characteristics and optimal surgical approach for these rare lesions remain areas of ongoing investigation, with laparoscopic techniques emerging as the preferred method for both diagnosis and treatment in appropriately selected cases.^4)^ We report the 10th case of falciform ligament lipoma and discuss 3 aspects that advance the understanding of this rare entity: (1) the role of MRI in preoperative anatomical characterization, including the umbilical vein remnant as a diagnostic landmark; (2) the utility of a systematic immunohistochemical panel for excluding malignant and borderline mimics; and (3) the safety and efficacy of laparoscopic excision with enhanced recovery in a large lesion.

## CASE PRESENTATION

### Patient information

A 49-year-old female presented to our surgical clinic with a chief complaint of chronic epigastric pain radiating to the right hypochondrium. The patient had been experiencing these symptoms for several years, with a recent exacerbation over the preceding months.

### Clinical findings

The patient reported chronic epigastric pain radiating to the right upper quadrant, which had been ongoing with periods of remission followed by recurrence. There were no associated symptoms such as nausea, vomiting, fever, or weight loss. Physical examination was unremarkable with no palpable mass or organomegaly. Notably, there were no signs of acute abdomen, which can occur in cases of lipoma torsion and infarction, as reported in the literature.^3)^

### Diagnostic assessment

Prior to imaging, the patient underwent an upper gastrointestinal endoscopy, which revealed no evidence of peptic ulcer disease, gastritis, or other mucosal pathology. Abdominal ultrasonography showed no gallstones, biliary dilatation, or liver parenchymal abnormality. Laboratory investigations, including liver function tests, serum amylase, and lipase, were within normal limits. Given the absence of any explanation for the chronic epigastric pain on these initial investigations, cross-sectional imaging with MRI was performed, which identified a falciform ligament mass.

### MRI findings

**Fig. 1:**
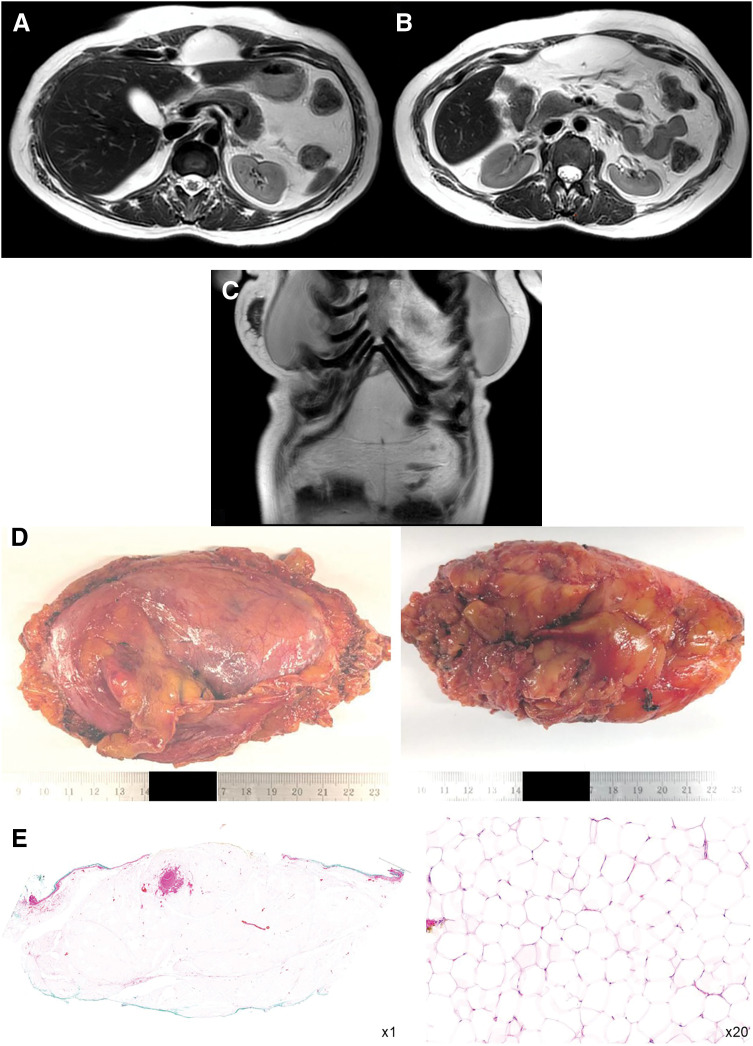
MRI of the falciform ligament lipoma. (**A**) Axial T2-weighted MRI at the level of the hepatic hilum demonstrating a large, well-defined fatty mass in the epigastric region. The lesion displays homogeneous signal intensity consistent with mature adipose tissue, with no septal thickening, nodular components, or internal enhancement. (**B**) Axial T2-weighted fat-suppressed MRI at a more caudal level showing the intimate anatomical relationship of the mass with the left hepatic lobe and the transverse colon, without evidence of parenchymal invasion. (**C**) Coronal T2-weighted fat-suppressed MRI demonstrating the full craniocaudal extent of the mass, spanning from the xiphoid process to the umbilicus and traversed by the remnant of the umbilical vein. The coronal view confirms the falciform ligament origin and the absence of hepatic parenchymal involvement. (**D**) Gross pathological appearance of the resected specimen. Left: Ventral surface of the well-encapsulated fatty mass (11 × 8 × 2.5 cm) with the attached segment of the falciform ligament containing the ligamentum teres hepatis. The intact fibrous capsule and the ligamentous pedicle are clearly demonstrated. Right: Dorsal surface of the specimen after sectioning, revealing homogeneous yellowish adipose tissue with a lobulated cut surface and no areas of necrosis or hemorrhage, consistent with a benign lipoma. (**E**) Histopathological examination of the resected specimen. Left (×1): Whole-section low-power view demonstrating the overall architecture of the encapsulated lesion, composed entirely of mature adipose tissue with a thin fibrous capsule and no areas of necrosis. Right (×20): Higher-magnification view revealing uniform mature adipocytes without cytological atypia, lipoblasts, or mitotic figures, separated by thin fibrous septa with minimal vascularity, consistent with a benign lipoma.

The MRI revealed a large fatty mass measuring 9 × 8.5 × 2.7 cm in the epigastric region. The lesion demonstrated characteristic features of fatty tissue without septal thickening or nodular components, and showed no pathological enhancement after gadolinium administration. The mass was noted to be in contact with the left hepatic lobe and transverse colon, with extension from the xiphoid process cranially to the umbilicus caudally. The lesion was partially traversed by the remnant of the umbilical vein. An additional finding included an 11-mm slow-flow hepatic hemangioma, while the biliary tract and pancreas appeared normal (**Fig. 1A** and **1B**).

The imaging characteristics were consistent with a voluminous lipoma most likely arising from the falciform ligament. Notably, the lesion demonstrated homogeneous fat signal intensity on MRI, well-defined margins, and absence of contrast enhancement (**Fig. 1A**–**1C**)—features that help differentiate benign lipomas from other falciform ligament pathologies.^2,8)^

### Treatment

Following surgical evaluation, surgical excision was recommended based on 3 converging indications: (1) chronic symptomatic pain refractory to conservative management; (2) inability to obtain a definitive tissue diagnosis preoperatively, necessitating histopathological confirmation; and (3) the large size of the lesion, which warranted oncologic exclusion of well-differentiated liposarcoma and PEComa, particularly given the known malignant potential of rare falciform ligament tumors. Given the diagnostic uncertainty and the need for precise anatomical localization, a laparoscopic approach was chosen.

### Operative procedure

Laparoscopic exploration was performed using the Hasson technique, with 4 trocars placed (one 12-mm umbilical optical trocar, two 10-mm trocars, and one 5-mm trocar). Diagnostic laparoscopy confirmed that the mass originated from the falciform ligament and extended to the sub-xiphoid region, with intimate adhesion to the diaphragm but without involvement of the liver parenchyma or stomach. Adhesiolysis was performed using electrocautery and ultrasonic dissection, and the round ligament was divided between Hem-o-lok clips. The specimen was extracted through a mini-laparotomy, and a concurrent umbilical hernia was repaired by direct suture. Operative time was 160 min with minimal blood loss and no perioperative complications.

### Histopathological examination

Macroscopic examination revealed a well-encapsulated fatty mass measuring 11 × 8 × 2.5 cm with an attached segment of the falciform ligament (5.5 × 5 × 1.5 cm) (**Fig. 1D**, left). The cut surface showed homogeneous yellowish adipose tissue with a lobulated appearance and no areas of necrosis (**Fig. 1D**, right). Histological examination demonstrated a proliferation of mature adipocytes without cytological atypia or lipoblasts, separated by thin fibrous septa with minimal vascularity and no inflammatory infiltrate (**Fig. 1E**). On immunohistochemistry, MDM2 was negative, effectively excluding well-differentiated liposarcoma. HMB-45, smooth muscle actin (SMA), and melan-A were all negative, ruling out PEComa. Vimentin immunoreactivity was positive, consistent with mesenchymal origin. The final pathological diagnosis was benign lipoma of the falciform ligament. The apparent discrepancy between the MRI dimensions (9 × 2.7 × 8.5 cm) and surgical specimen measurements (11 × 8 × 2.5 cm) is explained by several factors: differences in measurement plane (axial MRI short-axis vs. expanded specimen dimensions), tissue relaxation and decompression following release from adjacent structures, and the inclusion of the attached falciform ligament pedicle in the specimen measurements. This type of size discrepancy between *in vivo* imaging and *ex vivo* pathology is well recognized in soft tissue tumors of fatty composition.

### Outcome and follow-up

The patient had an uncomplicated postoperative course and was discharged on POD 1, consistent with the enhanced recovery protocol employed at our institution. At 6-month follow-up, the patient reported complete resolution of her chronic epigastric pain. No complications or recurrence were noted at the time of last follow-up. Long-term surveillance was planned given the rarity of the condition and limited long-term data available in the literature.

## DISCUSSION

Falciform ligament lipoma is an exceptionally rare entity in abdominal surgery. A systematic search of PubMed using the terms “falciform ligament lipoma” and “ligamentum teres lipoma” (last searched March 2026) identified 9 previously reported cases since 1983, making the present case the 10th in the published literature.^3,4,9–12)^ This extreme rarity poses significant challenges for diagnosis and treatment planning. The clinical features, imaging findings, surgical management, and outcomes of all reported cases, including the present case, are summarized in **Table 1**.

**Table 1:** Summary of reported cases of falciform ligament and ligamentum teres hepatis lipoma

Case	Author (year)	Location	Age/sex	Clinical presentation	Tumor size	Imaging	Treatment	Outcome
1	Adamsen (1983)^9)^	Ligamentum teres	NR	Acute abdomen (torsion/infarction)	NR	None reported	Open surgery	NR
2	Honda et al. (1983)^1)^	Falciform ligament	NR	NR	NR	CT	Open surgery	NR
3	Bruneton et al. (1987)^8)^	Falciform ligament	NR	Incidental	NR	US, CT	Conservative	NR
4	Budarin (1988)^10)^	Ligamentum teres	NR	NR	Large (NR)	None reported	Conservative	NR
5	Farkas et al. (1991)^11)^	Ligamentum teres	NR	NR	Giant (NR)	None reported	Open surgery	NR
6	Kakitsubata et al. (1993)^2)^	Falciform ligament	NR	Abdominal mass	NR	US, CT, MRI	Open surgery	NR
7	Makama et al. (2016)^12)^	Falciform ligament	NR	Chronic epigastric pain	NR	US	Open surgery	NR
8	Bangeas et al. (2020)^3)^	Ligamentum teres	43F	Acute epigastric pain, nausea, vomiting (torsion + infarction)	2.8 × 1.3 cm	CT, MRI	Laparoscopic excision	Good; discharged POD 1
9	Sadeghi et al. (2021)^4)^	Falciform ligament	23F	Abdominal pain and distension (4 months)	28 × 23 × 28 cm	NR	Open surgery	NR
10	Present case (2026)	Falciform ligament	49F	Chronic epigastric pain radiating to the right hypochondrium (several years)	11 × 8 × 2.5 cm	MRI (T2, FS-T2)	Laparoscopic excision	Complete pain resolution at 6 months; no recurrence

Cases 1–7 are based on the literature table reported by Bangeas et al. (J Surg Case Rep. 2020). Cases 1 (Danish), 4 (Russian), and 5 (Hungarian) were published in non-English languages; detailed clinical data were not available in English-language sources. The present case (Case 10) is highlighted in blue.

F, female; FS-T2, fat-suppressed T2-weighted MRI; NR, not reported; US, ultrasonography

The clinical presentation is typically nonspecific, with chronic epigastric pain being the most frequently reported symptom across documented cases.^3,4)^ Tumor size varies dramatically across reports—from our lesion measuring 11 × 8 × 2.5 cm to the 28 × 23 × 28 cm giant tumor reported by Sadeghi et al.^4)^—and this likely accounts for variation in symptom severity. Although chronic presentation is most common, acute complications such as torsion and infarction mimicking acute abdomen have also been documented^3)^; therefore, this entity should be considered across a broad spectrum of clinical scenarios.

Cross-sectional imaging, particularly MRI, plays a central role in preoperative evaluation. Characteristic features include homogeneous fatty signal intensity, well-defined margins, and absence of contrast enhancement.^1,2)^ On CT, pure lipomas typically demonstrate attenuation values of −65 to −120 Hounsfield units, while on US they appear as hyperechoic, well-circumscribed lesions with posterior acoustic attenuation.^8)^ In our case, the umbilical vein remnant traversing the mass and its anatomical continuity with the falciform ligament were clearly depicted on MRI, facilitating preoperative planning. US typically demonstrates these lesions as well-defined, hyperechoic masses; however, as in our case, it may fail to identify the lesion when it is positioned in a deep retroperitoneal or subxiphoid location. CT allows rapid assessment and is particularly useful for emergency presentations such as torsion or infarction, as well as for assessing adjacent organ involvement; however, it carries radiation exposure and provides less soft-tissue contrast than MRI for characterization of complex fatty lesions. MRI is therefore the preferred modality for elective evaluation, as fat-specific sequences (T1 in-phase/out-of-phase, fat-suppressed T2) allow precise characterization of lesion composition and anatomical relationships, as demonstrated in our case. Despite these characteristic features, imaging alone cannot always yield a definitive diagnosis for peritoneal-based masses, and histopathological confirmation remains essential.

The differential diagnosis has expanded significantly with increasing recognition of rare falciform ligament tumors. PEComas, including clear cell myomelanocytic tumor—a subtype that most frequently arises from the falciform ligament—are characterized by expression of both smooth muscle and melanocytic markers (HMB-45, SMA, melan-A) and show arterial-phase enhancement distinct from the non-enhancing pattern of lipomas.^5–7)^ Malignant neoplasms, particularly liposarcoma, must also be excluded; MDM2 immunostaining is essential for this purpose, as it is positive in liposarcomas but negative in benign lipomas.^13)^ Other entities to consider include low-grade fibromyxoid sarcoma,^14)^ solitary fibrous tumor,^15)^ leiomyosarcoma,^16)^ twisted lipomatous appendages,^17)^ falciform ligament cysts,^18)^ and lymphangioma.^19)^ In our case, negative MDM2, HMB-45, SMA, and melan-A staining effectively excluded both liposarcoma and PEComa, confirming the benign nature of the lesion. In the present case, the absence of arterial enhancement and internal nodularity on MRI effectively narrowed the differential toward a benign lipomatous lesion preoperatively, while the systematic 4-marker immunohistochemistry panel provided definitive postoperative confirmation—a combined approach we recommend for future cases of falciform ligament masses.

Given the limitations of imaging in characterizing rare falciform ligament masses, diagnostic laparoscopy plays an important complementary role.^4)^ In our case, laparoscopic exploration confirmed the falciform ligament origin and the precise anatomical relationships of the mass prior to resection, which proved critical for surgical planning. Complete laparoscopic excision is the treatment of choice for symptomatic lesions, offering excellent visualization, minimal invasiveness, and favorable recovery.^4)^ Open surgery may be required for extremely large tumors or when laparoscopic expertise is unavailable.^4)^ Comprehensive histopathological evaluation—including assessment for mature adipocytes without atypia, absence of lipoblasts, and a targeted immunohistochemical panel—is essential to confirm the diagnosis and exclude malignancy. ^5–7,13)^

The prognosis following complete excision is excellent. Our patient experienced complete resolution of chronic epigastric pain by the 6-month follow-up, with no evidence of recurrence. Although long-term data remain limited due to the rarity of this condition,^4)^ the risk of recurrence after complete resection of a benign lipoma is considered minimal.

## CONCLUSIONS

We report the 10th case of falciform ligament lipoma in the published literature. Although rare, this entity should be included in the differential diagnosis of chronic epigastric pain. Comprehensive immunohistochemical analysis—including MDM2, HMB-45, SMA, and melan-A—is essential to exclude liposarcoma and PEComa. ^5–7,13)^ Complete laparoscopic excision is considered the treatment of choice, with excellent symptomatic outcomes and minimal risk of recurrence.^4,14)^

## DECLARATIONS

### Availability of data and materials

Not applicable. This is a case report and does not involve any datasets generated or analyzed during the study beyond the patient’s clinical data, which are not publicly available to protect patient privacy.
